# Towards good correlation between fragment molecular orbital interaction energies and experimental IC_50_ for ligand binding: A case study of p38 MAP kinase

**DOI:** 10.1016/j.csbj.2018.10.003

**Published:** 2018-10-13

**Authors:** Yinglei Sheng, Hirofumi Watanabe, Keiya Maruyama, Chiduru Watanabe, Yoshio Okiyama, Teruki Honma, Kaori Fukuzawa, Shigenori Tanaka

**Affiliations:** a Graduate School of System Informatics, Kobe University, 1-1 Rokkodai, Nada-ku, Kobe 657-8501, Japan; b Education Center on Computational Science and Engineering, Kobe University, 7-1-48 Minatojimaminamimachi, Chuo-ku, Kobe 650-0047, Japan; c Center for Biosystems Dynamics Research, RIKEN, 1-7-22 Suehiro-cho, Tsurumi-ku, Yokohama, Kanagawa 230-0045, Japan; d Division of Medicinal Safety Science, National Institute of Health Sciences, 3-25-26 Tonomachi, Kawasaki-ku, Kawasaki 210-9501, Japan; e Department of Physical Chemistry, School of Pharmacy and Pharmaceutical Sciences, Hoshi University, 2-4-41 Ebara, Shinagawa, Tokyo 142-8501, Japan

**Keywords:** Ab initio calculation, FMO method, p38 MAP kinase, Ligand binding affinity, In silico screening

## Abstract

We describe several procedures for the preprocessing of fragment molecular orbital (FMO) calculations on p38 mitogen-activated protein (MAP) kinase and discuss the influence of the procedures on the protein–ligand interaction energies represented by inter-fragment interaction energies (IFIEs). The correlation between the summation of IFIEs for a ligand and amino acid residues of protein (IFIE-sum) and experimental affinity values (IC_50_) was poor when considered for the whole set of protein–ligand complexes. To improve the correlation for prediction of ligand binding affinity, we carefully classified data set by the ligand charge, the DFG-loop state (DFG-in/out loop), which is characteristic of kinase, and the scaffold of ligand. The correlation between IFIE-sums and the activity values was examined using the classified data set. As a result, it was confirmed that there was a selected data set that showed good correlation between IFIE-sum and activity value by appropriate classification. In addition, we found that the differences in protonation and hydrogen orientation caused by subtle differences in preprocessing led to a relatively large difference in IFIE values. Further, we also examined the effect of structure optimization with different force fields. It was confirmed that the difference in the force field had no significant effect on IFIE-sum. From the viewpoint of drug design using FMO calculations, various investigations on IFIE-sum in this research, such as those regarding several classifications of data set and the different procedures of structural preparation, would be expected to provide useful knowledge for improvement of prediction ability about the ligand binding affinity.

## Graphical abstract

**Figure:**
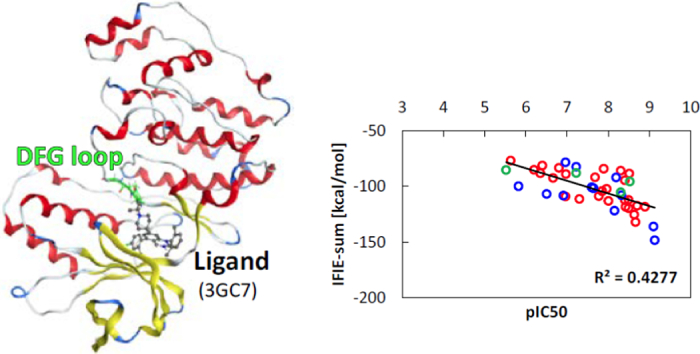

## 1 Introduction

**Fig. 1:**
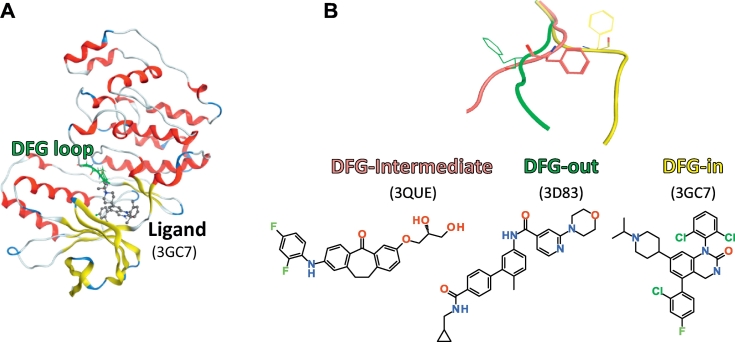
(A) Complex of the p38 MAP kinase and its ligand, where the ribbon and ball-and-stick modes correspond to the protein and ligand, respectively. (B) DFG-in, DFG-out, and DFG-intermediate loop structures shown as yellow, green, and pink tubes, respectively.

The fragment molecular orbital (FMO) method [[1], [2], [3]] allows for thequantum mechanical study of large biomolecules. It provides not only accurate protein–ligand interactions but also their energetic components for each fragment pair, which is called the inter-fragment interaction energy (IFIE). The FMO method was applied to several protein–ligand systems such as nuclear receptors, kinases, proteases, and protein–protein interaction systems, which are promising drug targets [[4], [5], [6], [7], [8], [9], [10], [11], [12]]. The application to the estrogen receptor–ligand binding system [4], for example, shows a good correlation between the binding energy and experimental relative binding affinity. However, for the complex structure used as the input for the FMO calculation, it has not been sufficiently studied how to prepare the structure, including the addition of hydrogen atoms, the necessity of structure optimization, and the selection of force field. Therefore, in this study, to find a relevant prescription of structure preparation suitable for the bioactivity prediction with FMO method, the structures were prepared according to several protocols and consequently the IFIEs were estimated. This is the first example to see the correlation of (IFIE-sum) obtained from various structures including the differences of the ligand charges and/or tertiary structures for the same target protein. In our experience, it is often difficult to observe that the IFIE-sum and bioactivity values are completely or successfully correlated using all of the calculated data. Therefore, the correlation is investigated while carefully trying to classify the data according to charge state of the ligand, skeleton and tertiary structure of the protein. To evaluate the capability of FMO approach for binding-affinity prediction and the influence of structure preparation protocol on it, we chose the p38 mitogen-activated protein (MAP) kinase [[13], [14], [15]] (Fig. 1A) as a target protein. The reason is that this protein has many entries of X-ray crystal structures and experimental activity data; the 95 structures with IC_50_ data in the ChEMBL database [16] were available in the Protein Data Bank (PDB) [17].

In this paper, we analyze 78 PDB structures because the FMO calculations for these structures successfully converged and provided IFIE values, while the calculations for the other structures did not converge for some structural reasons. Because all inhibitors which we dealt with have corresponding PDB structures and are placed in the same binding pocket, these inhibitors are specific inhibitors. Thus, we denote these inhibitors as ‘ligands’. We also regard the IC_50_ value as the measure of ligand binding affinity in this study. The kinases play important roles in the functional expression of various cellular processes, which are involved in cell aging and autoimmune diseases, and are activated by phosphorylation under external stress such as heat, osmotic pressure and ultraviolet radiation [13]. It is well known that the p38 MAP kinase has two main stable structures: the DFG (Asp-Phe-Gly)-in-loop and the DFG-out-loop forms (Fig. 1B). In addition, several structures include a DFG-intermediate-loop form in our dataset (Fig. 1B).

In the following sections, the preparation protocols are first described in Sec. 2. Next, we evaluated IFIEs between the p38 MAP kinase and its various inhibitors using the FMO method, and compared the IFIE-sum with experimental data in Sec. 3.1. We also categorized the proteins in terms of their DFG-in/out-loop configuration and the ligands according to their scaffold. We then evaluated the difference in IFIEs with and without complementation of missing residues. Additionally, we investigated the difference in IFIEs when different force fields based on molecular mechanics (MM) model were used to optimize the complexes. These results are presented in Sec. 3.2.

## 2 Materials and computational methods

**Fig. 2:**
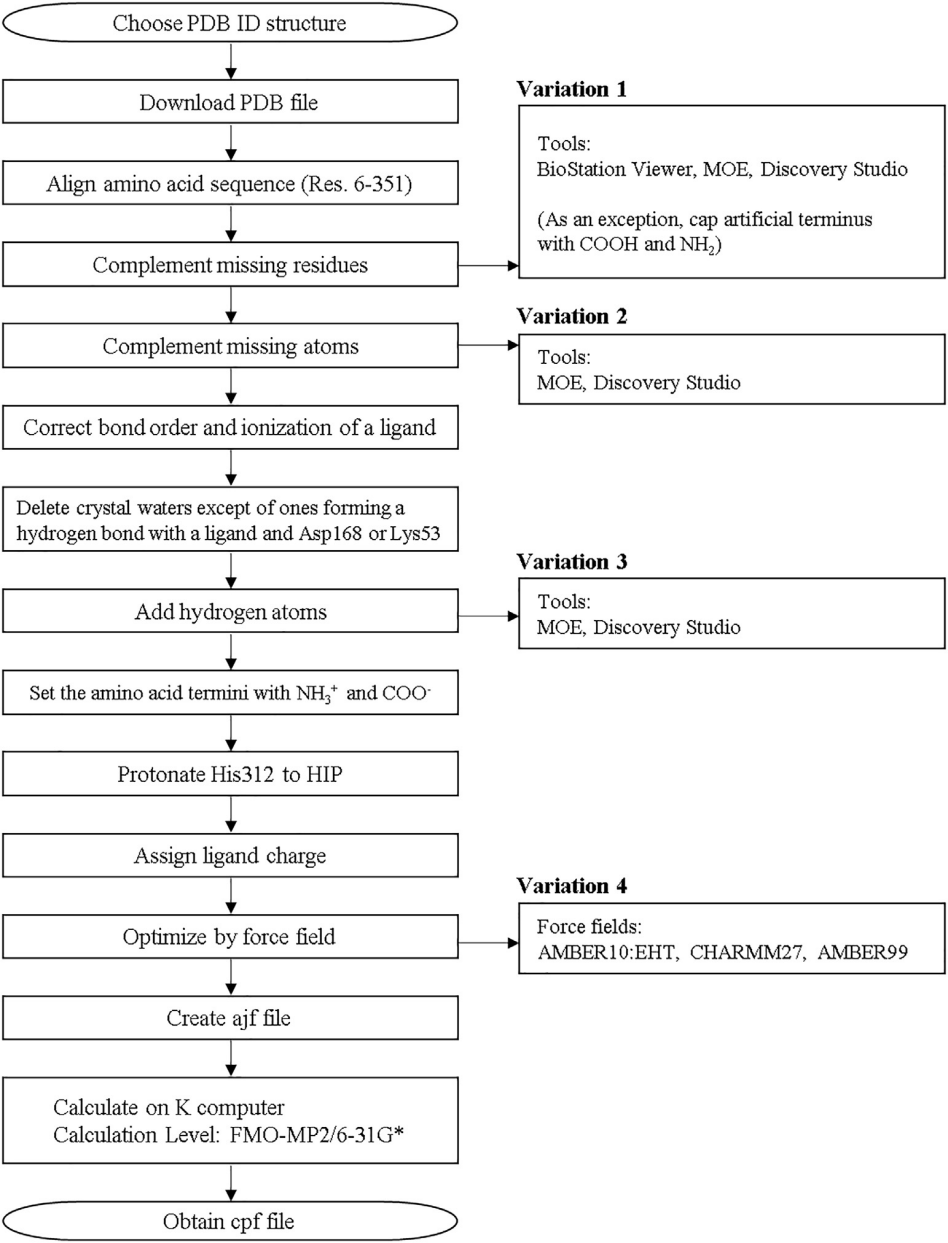
Flow chart of the manual modeling and FMO calculation protocol.

**Fig. 3:**
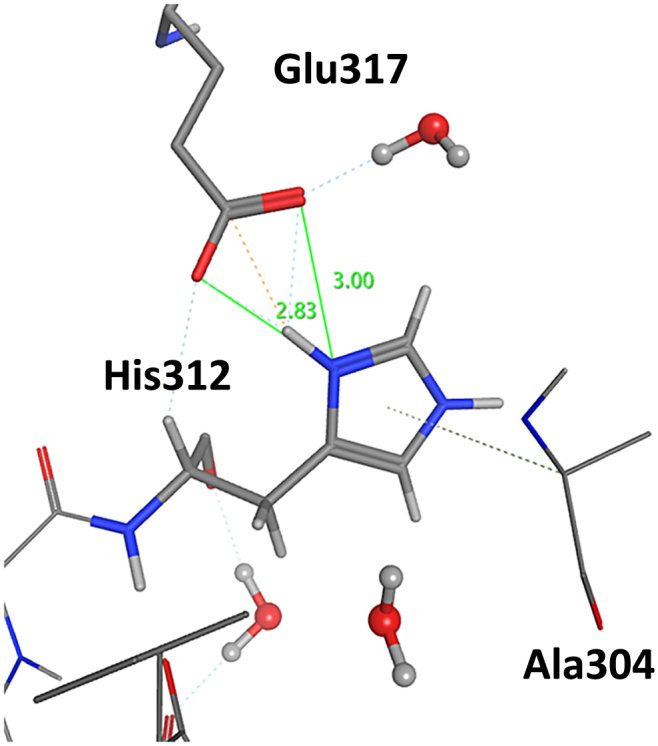
3D structure around His312. As Glu317 forms a salt bridge with His312, the doubly-protonated histidine, HIP, was assigned to this residue by Protonate3D in MOE. Numerals denote the interatomic distances in units of Å.

The structures of p38 MAP kinase–ligands complexes used in this study were prepared and processed according to the protocols below (summarized in Fig. 2). First, the X-ray structures of the complexes were downloaded from the PDB repository [17]. If the protein structure has missing atoms, they were completed with every possible missing side-chain and main-chain atoms. For the missing main chain atoms, the “Complement Main Chain” function of the BioStation Viewer [3] was employed. In this process, we employed PDB ID: 3GC7 for the DFG-in conformation and 3D83 for the DFG-out conformation, respectively, as template structures because they have the best resolution and no missing residues. For the side chain atoms, the “Structure Preparation” function in Molecular Operation Environment (MOE) [18] was employed. All crystallographic water molecules were removed except for the following cases: the water molecule forming a hydrogen bond with Asp168 or Lys53 in the DFG-in conformation and the water molecule forming a hydrogen bond with Asp168 in the DFG-out conformation, because the numbers of crystallographic water molecules in binding pocket were different for each PDB structure. Sometimes the molecular modeling software fails to assign the bond order of ligands, which in turn fails to assign the correct protonation state of the ligand. When this issue arose, we manually corrected the bond order of the ligand. We carefully addressed this issue because a difference in protonation state frequently leads to large differences in IFIEs. The “Protonate3D” function [19] in MOE and GBpK [20] in Discovery Studio were employed for the determination of protonation states. All histidine residues except for His312 were set to have neutral charges. For His312, we chose the HIP state, which is positively charged state and has hydrogen atoms on both nitrogen atoms of the imidazole ring, for all complexes because Protonate3D provided a charged state for this residue for more than 50% of the representative structures and the side chain of His312 forms a salt bridge with Glu317, as shown in Fig. 3. The positions of the hydrogen atoms and of the added atoms were optimized with three classical force fields, AMBER10:EHT, CHARMM27, and AMBER99, where AMBER10:EHT is a typically used force field. The 78 structures were distributed among the four research groups of different institutions which contributed to the study for preparation and optimization, and each group used a different modeling tool, where PDB structures for one ligand type were dealt with by multiple institutions. We made 6 structure sets of A, B, C, C′, D and D' containing 38, 8, 25, 25, 15 and 15 complexes, respectively (see Table 1), where several complexes overlapped between the sets. Each institution did not have the same modeling tool; thus, preprocessing before the FMO calculations was performed in slightly different ways. Structures A, C, and D were named by each modeling tool. Differences were introduced in each step owing to the different tools employed for the determination of the protonation state, the force fields used for structure optimization, and the way that the missing residues were added. In addition, as these differences were concurrently introduced, we could not distinguish the effect of the change in a single step. Thus, to evaluate the effect of such differences on the IFIE values, we built Structures C′ and D′ to evaluate the influence of the force field, and Structure B to evaluate the effect of the added missing residues. Details of the preparation procedure for each structure are provided in the Appendix.

**Table 1:** Details of preprocess for each structure type.^a^

Structure	# of complex	Research group^b^	Force field	Complementation of missing residues	Complementation of missing atoms	Addition of hydrogen atoms	Optimization	Modeling software
A	38	Institution a.	AMBER10:EHT	BioStation Viewer with template structures.	MOE with “Structure Preparation” function.	MOE with “Protonate3D” function.	MOE for complemented heavy atoms and all hydrogen atoms.	BioStation Viewer MOE
B	8	Institution a.	AMBER10:EHT	Capping artificial terminus with COOH and NH_2_ by MOE instead of complementation.	MOE with “Structure Preparation” function.	MOE with “Protonate3D” function.	MOE for all hydrogen atoms.	BioStation Viewer MOE
C	25	Institutions b and c.	CHARMM27	BioStation Viewer or Discovery Studio with template structures.	Discovery Studio with “Clean Protein” function.	Discovery Studio with “GBpK” function.	Discovery Studio for complemented heavy atoms and all hydrogen atoms.	BioStation Viewer Discovery Studio
C′	25	Institutions b and c. Only optimization by institution a.	AMBER10:EHT	BioStation Viewer or Discovery Studio with template structures.	Discovery Studio with “Clean Protein” function.	Discovery Studio with “GBpK” function.	MOE for all hydrogen atoms.	Discovery Studio MOE
D	15	Institution d.	AMBER99	BioStation Viewer or MOE with template structures.	MOE with “Structure Preparation” function.	MOE with “Protonate3D” function.	MOE for complemented heavy atoms and all hydrogen atoms.	BioStation Viewer MOE
D′	15	Institution d. Only optimization by institution a.	AMBER10:EHT	BioStation Viewer or MOE with template structures.	MOE with “Structure Preparation” function.	MOE with “Protonate3D” function.	MOE for all hydrogen atoms.	BioStation Viewer MOE

a N- and C-termini are in the form of NH_3_^+^ and COO^-^, respectively, in all the structures.

b Four institutions, a, b, c and d, were involved in structure preparation, calculation and analysis.

We performed FMO calculations using ABINIT-MP [3,21,22] version 6+ and evaluated the inter-fragment interaction energies (IFIE) as

(1) $$\Delta {{\tilde{E}}^{\prime }}_{IJ}={E^{\prime }}_{IJ}-{E^{\prime }}_{I}-{E^{\prime }}_{J}+\text{Tr}\left(\Delta D^{IJ}V^{IJ}\right)$$

where *E*^′^_*X*_ is the monomer *(X = I, J)* or dimer *(X = IJ)* energy without the environmental electrostatic energy, *V*^*IJ*^ is the electrostatic potential of the fragment pair *IJ*, and Δ*D*^*IJ*^ is the difference density matrix. The FMO-MP2/6-31G* [3,23] level was employed for the calculations. We treated each amino acid residue as one fragment and the ligand as well. The IFIE-sum was defined as the sum of the IFIE between a ligand and an amino acid residue, and the IFIE between the ligand and a water molecule was not included in the IFIE-sum. We used the K computer (in Kobe, Japan) for the FMO calculations. An example of calculation time was 8.6 h (for PDB ID: 1BL6), which mainly depended on the number of atomic orbitals in the ligand fragment.

## 3 Results and discussion

### 3.1 Correlation between pIC_50_ and ligand binding interaction energies

#### 3.1.1 Classification of proteins in terms of DFG-in/out-loop conformation

**Fig. 4:**
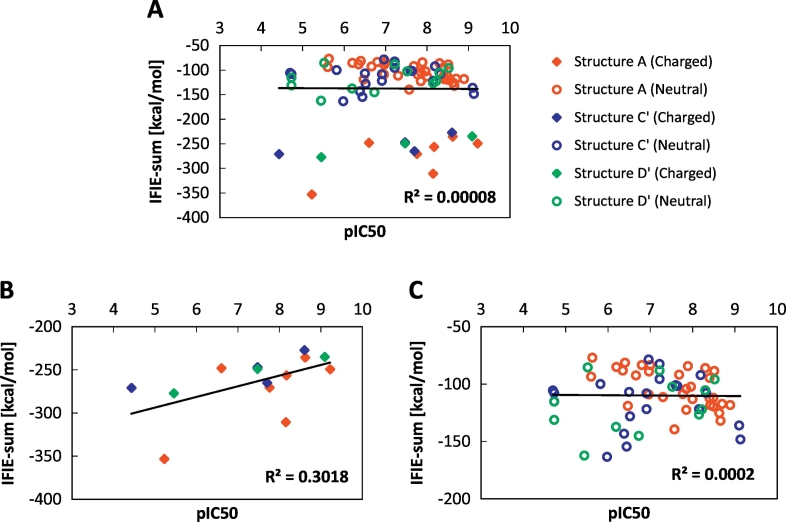
Correlation between the pIC_50_ and predicted binding energies (IFIE-sums) for (A) all the structures, (B) structures with charged ligands, and (C) structures with neutral ligands. These figures are obtained from structures A (red), C′ (blue), and D′ (green) including DFG-in, DFG-intermediate, and DFG-out proteins. The neutral and charged ligands are marked with circle and diamond, respectively.

First, we evaluated the correlation between the experimental IC_50_ values and the calculated IFIEs for 78 complexes for Structures A, C′ and D', where these structures were optimized by the same force field (AMBER10:EHT). Fig. 4A shows the relationship between the pIC_50_ and IFIE-sums of all complexes with unique 78 compounds, which has a low correlation coefficient of *R*^2^ = 0.00008. Because the difference in IFIE-sum between the neutral and charged ligands is more than 100 kcal/mol, as shown in Fig. 4A, it is difficult to collectively compare these data. Thus, we investigated the correlation between the pIC_50_ and IFIE-sums for ligands with similar net charges. Generally, comparing the IFIE values of neutral and charged fragments was difficult because the absolute value of the IFIE for charged fragment pairs was overestimated. Fig. 4B and C show the correlation between the pIC_50_ and IFIE-sums for complexes with charged and neutral ligands, respectively. Even when separately considering the neutral and charged ligands, low and nearly no correlations were obtained, respectively.

**Fig. 5:**
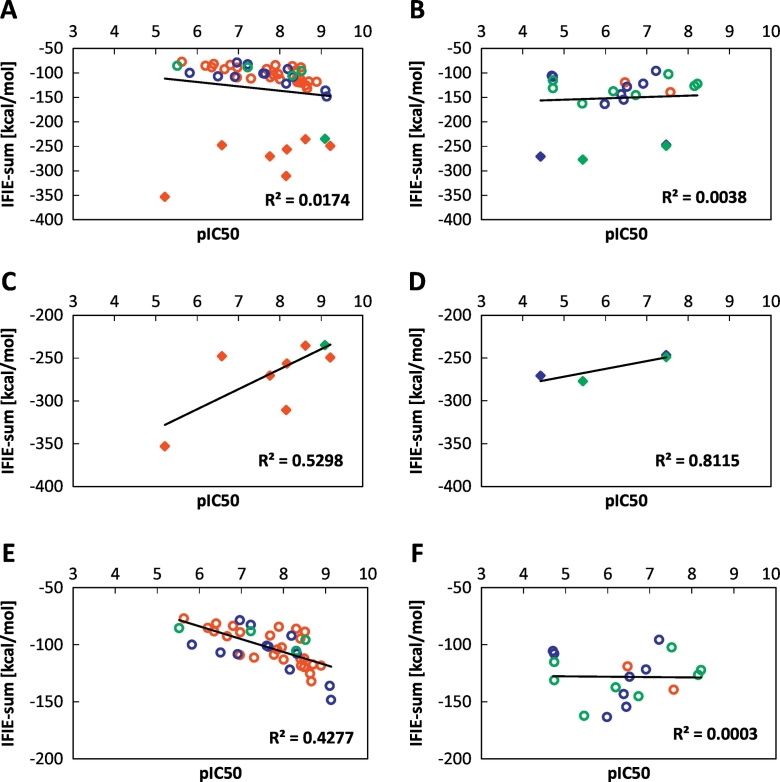
Correlation between the pIC50 and predicted binding energies (IFIE-sums) for DFG-in conformation and DFG-out conformation with different ligand scaffolds: (A) combined ligands with DFG-in proteins, (B) combined ligand with DFG-out proteins, (C) charged ligands with DFG-in proteins, (D) charged ligands with DFG-out proteins, (E) neutral ligands with DFG-in proteins, and (F) neutral ligands with DFG-out proteins. These figures are obtained from structures A (red), C′ (blue), and D' (green). The neutral and charged ligands are marked with circle and diamond, respectively.

Second, we classified the proteins in terms of their two characteristic conformations: the DFG-in-loop and DFG-out-loop (Fig. 5). Fig. 5A and B show the relationship between the pIC_50_ and IFIE-sums of the DFG-in and DFG-out conformations, respectively. In both cases, the IFIE-sums are not correlated with the pIC_50_. Additionally, these results were separated according to ligand charge, as shown in Fig. 5C–F. The IFIE-sums of the charged ligands show inversed correlations with the pIC_50_ for both the DFG-in and DFG-out conformations (Fig. 5C and D). However, the DFG-in conformation with neutral ligands shows a good correlation (*R*^2^ = 0.4277), as seen in Fig. 5E. On the other hand, no correlation was obtained (*R*^2^ = 0.0003) for the DFG-out conformation with neutral ligands, as shown in Fig. 5F. Such a poor correlation for the DFG-out structures would be caused by strong interaction between the ligand and Glu71 because experimental IC_50_ values do not always represent significant inhibition when the IFIE value between ligand and Glu71 indicates strong stabilization [24]. In the next section, we investigate the correlation for the neutral ligands, and the relationship between the pIC_50_ and IFIE-sums could be understood in terms of ligand scaffold.

#### 3.1.2 Categorizing in terms of ligand scaffold

**Fig. 6:**
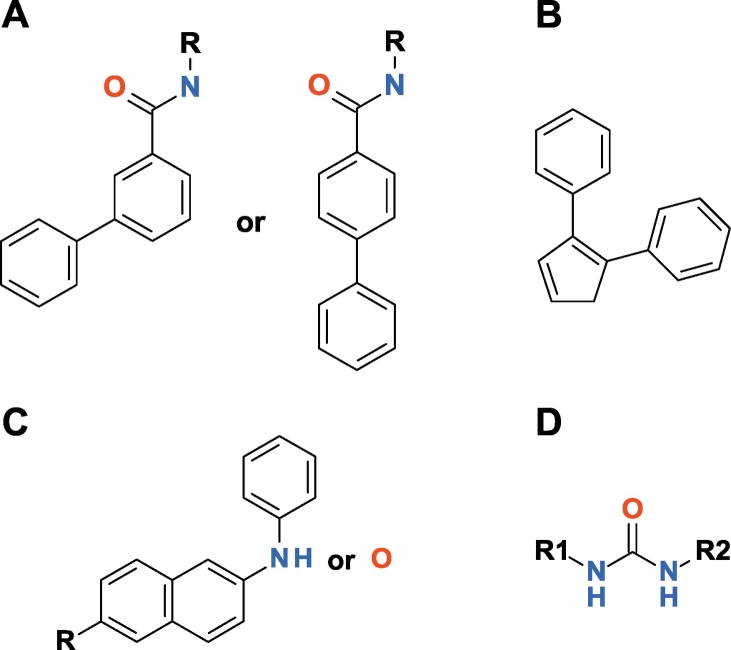
Ligand types: (A) biphenyl amides, (B) three linked aromatic rings, (C) fused aromatic rings with —NH— or —O— links, and (D) ureas. (E) Others are not depicted because there is no common scaffold in this type of ligands.

The correlation between the experimental pIC_50_ and the IFIE-sums of 64 neutral ligands (where there are 64 PDB structures with neutral ligands in 78 calculated PDB structures) is shown in Fig. 4C. Contrary to our expectations, there is no correlation between the experimental pIC_50_ value and the calculated IFIE-sum values for these ligands (*R*^2^ = 0.0002). However, the IFIE-sums of the p38 MAP kinase in the DFG-in conformation with 42 neutral ligands were significantly correlated to the pIC_50_ values, as shown in Fig. 5E. To understand the origin of this relationship, we divided the 64 neutral ligands into five categories based on the scaffold of the ligand by visual inspection, where the proteins were not separated by the DFG-loop conformation. The five types are defined as follows: (A) biphenyl amides, (B) three linked aromatic rings, (C) fused aromatic rings, (D) ureas, and (E) others (see Fig. 6).

**Fig. 7:**
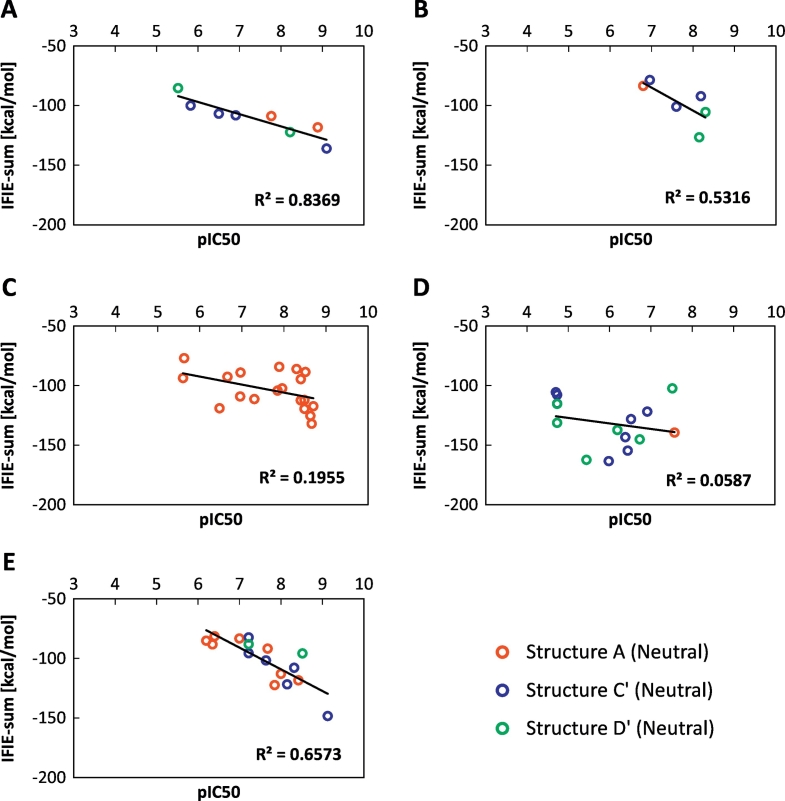
Correlation between the calculated IFIE-sum and the experimentally measured pIC_50_ for each ligand scaffold: (A) biphenyl amides, (B) three linked aromatic rings, (C) fused aromatic rings with —NH— or —O— links, (D) ureas, and (E) others. These figures are obtained from structures A (red), C′ (blue), and D' (green).

Fig. 7 shows the relationship between the pIC_50_ and IFIE-sums for each ligand type. As shown in Fig. 7A, B, C, and E, the IFIE-sums exhibited good correlations with pIC_50_. On the other hand, the correlation coefficient was extremely poor for urea-type ligands. The X-ray crystal structures showed that all complexes of urea ligands and p38 MAP kinases consisted of DFG-out-loop structures. This data has a behavior similar to that of the data in Fig. 5F, which presents the correlation between the pIC_50_ and IFIE-sums of the DFG-out proteins with neutral ligands. In our current dataset, the type of ligand scaffold has its preferable protein conformations for DFG-loop.

### 3.2 Influence of structure preparation on the IFIE values

#### 3.2.1 Influence of the complementation procedure for missing residues

**Fig. 8:**
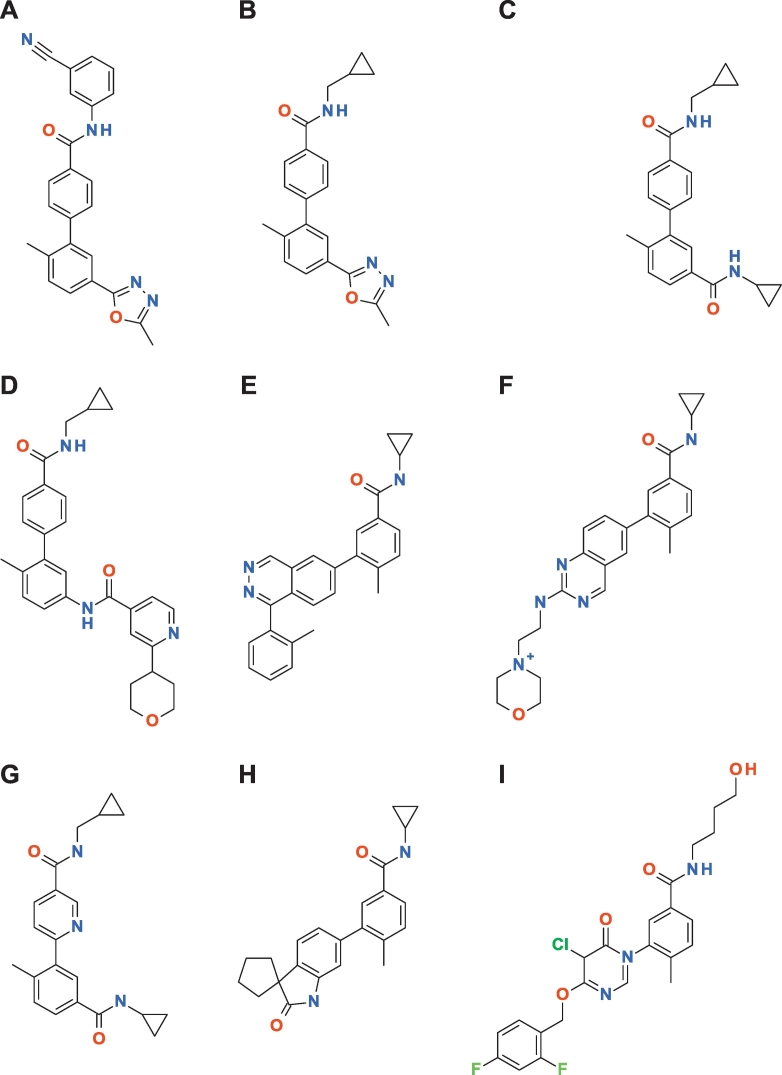
Structure of the biphenyl amide ligands for various PDB codes: (A) 2ZB0, (B) 2ZB1, (C) 3D7Z, (D) 3D83, (E) 3DS6, (F) 3DT1, (G) 3IPH, (H) 3RIN, and (I) 3ROC.

In this section, we describe the influence of the preparation of the structures on the IFIE values. For this purpose, we employed the biphenyl amide ligands as an example dataset, which included nine entries with PDB IDs: 2ZB1, 3D7Z, 3D83, 3DT1, 3IPH, 3RIN, 3ROC, 2ZB0, and 3DS6. Because the ligand in 3DT1 was a charged species, we only considered the neutral ligands for the discussion (Fig. 8).

#### 3.2.2 Complementation of missing residues

**Fig. 9:**
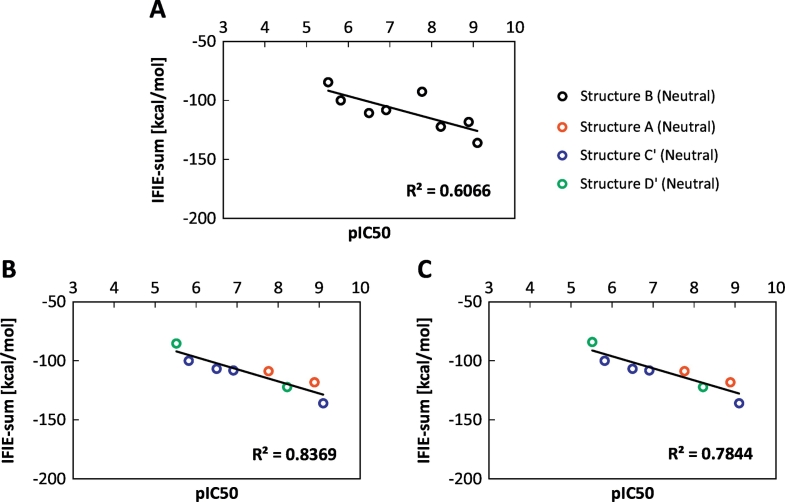
Correlation between the pIC_50_ and IFIE-sum for biphenyl amide ligands: (A) IFIE-sum for non-complemented structures, (B) IFIE-sum in the case of complemented structures where summation of IFIE values were taken for whole residues in the protein, and (C) IFIE-sum in the case of complemented structures where summation of IFIE values were taken with excluding main-chain complemented residues. Fig. A are obtained from structures B (black) and Figs. B and C are obtained from A (red), C′ (blue), and D' (green) including DFG-in, DFG-intermediate, and DFG-out proteins.

First, we investigate the treatment of the missing residues. Occasionally, the original structure from the PDB repository has missing residues and atoms, and there are two possible ways to treat the missing sections: (i) by adding the missing residues following a template and (ii) by capping the termini. We compared the results obtained with both procedures, as shown in Fig. 9. The correlation between IFIE-sum and pIC_50_ based on non-complemented and complemented structures is illustrated in Fig. 9A and B, respectively. Furthermore, to separate the effects of complementation, we evaluated IFIE-sums with excluding the IFIE values of missing residues using the complemented structure data shown in Fig. 9C. Note that the employed structures are identical in Fig. 9A and C and the IFIE-sum both in Fig. 9B and C contains only the contribution from the non-complemented region while that in Fig. 9A also contains the contribution from the complement region. The squared correlation coefficient *R*^2^ of the complemented structure was changed by 0.23 compared with the value from the non-complemented structure. This difference can be separated into two contributions such as the IFIE values from complemented residues (in missing region) and the changes (caused by the complementation) of IFIE values from non-missing region. Surprisingly, Fig. 9C shows the latter contribution was larger than the former.

#### 3.2.3 Hydrogen orientation and protonation state

**Fig. 10:**
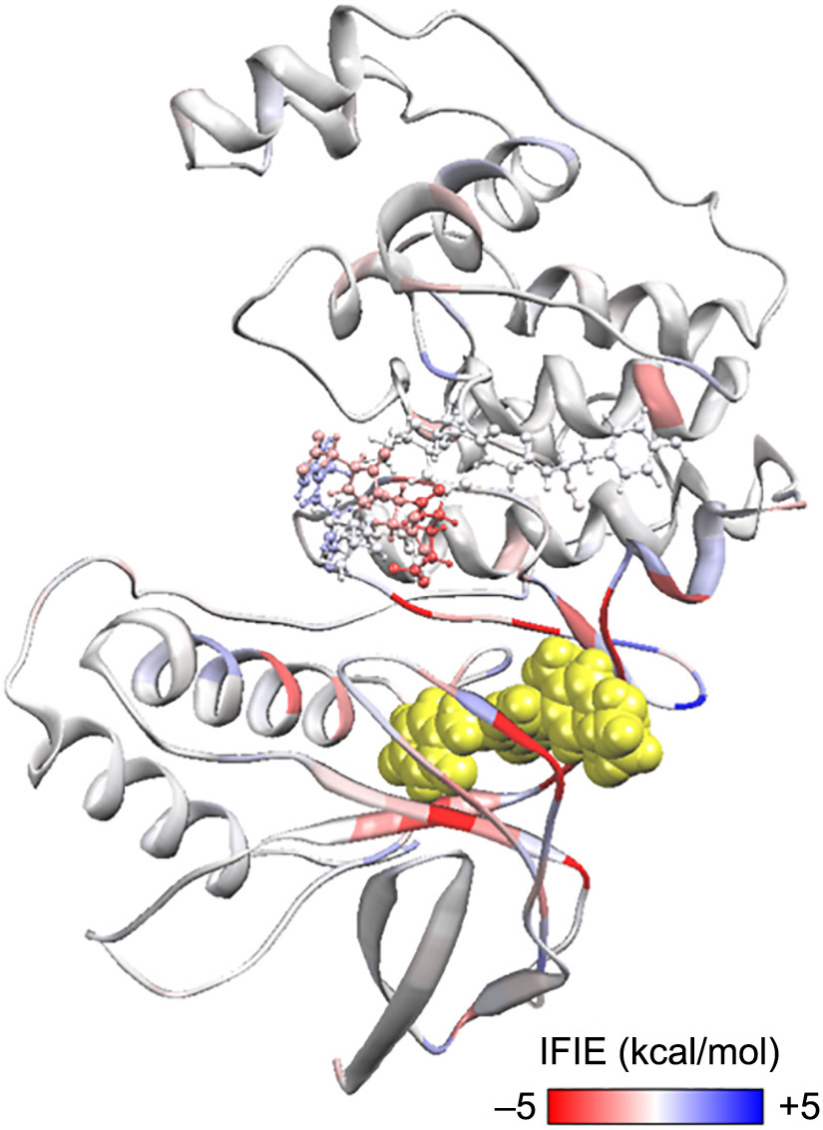
Ribbon representation of the structure which was built by BioStation Viewer with starting from the 3ROC structure in PDB. The red residues show attraction against the ligand (yellow) and the blue residues show repulsion. The green colour indicates the missing residues. The distance from the ligand to these missing residues was 10 Å or more even at the closest residues (residue number 172–182).

In this section, we investigate the differences in IFIE values of complemented and non-complemented structures using 3ROC as an example (Fig. 10). In the non-complemented structures, the N-terminus and C-terminus near the incomplete region were capped with NH_2_ and COOH, respectively. The complemented structures were built with template structure (PDB ID: 3GC7) using BioStation Viewer and Discovery Studio. The IFIE-sums of the complemented and the non-complemented structures were - 108.8 and − 92.6 kcal/mol (data not shown), respectively. Table 2 displays the IFIE of ligand with the complemented residues of the structure A (PDB ID: 3ROC). The sum of IFIE values of the complemented region was only −5.31 kcal/mol, but the difference in the IFIE-sum of the complemented and non-complemented structures was −16.2 kcal/mol [= (−108.8 + 92.6) kcal/mol].

**Table 2:** IFIE (in kcal/mol) between the ligand and complemented residues in the missing region (PDB ID: 3ROC).

Complemented residue	IFIEs with ligand [kcal/mol]
Ala172	0.27
Arg173	1.84
His174	−0.23
Thr175	0.39
Asp176	−1.84
Asp177	−2.33
Glu178	−4.07
Met179	0.02
Thr180	0.17
Gly181	0.12
Tyr182	0.34
IFIE-sum	−5.31

**Fig. 11:**
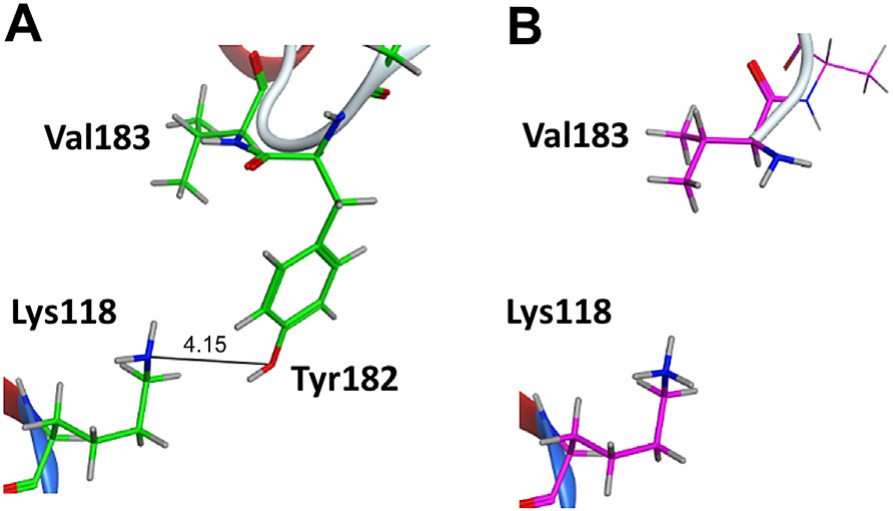
Comparison of the 3D structure of the complemented and non-complemented regions near the Lys118, Tyr182, and Val183 residues (PDB ID: 3ROC): (A) complemented structure and (B) non-complemented structure. The side chain of Lys118 was protonated in the non-complemented structure but not in the complemented structure.

**Fig. 12:**
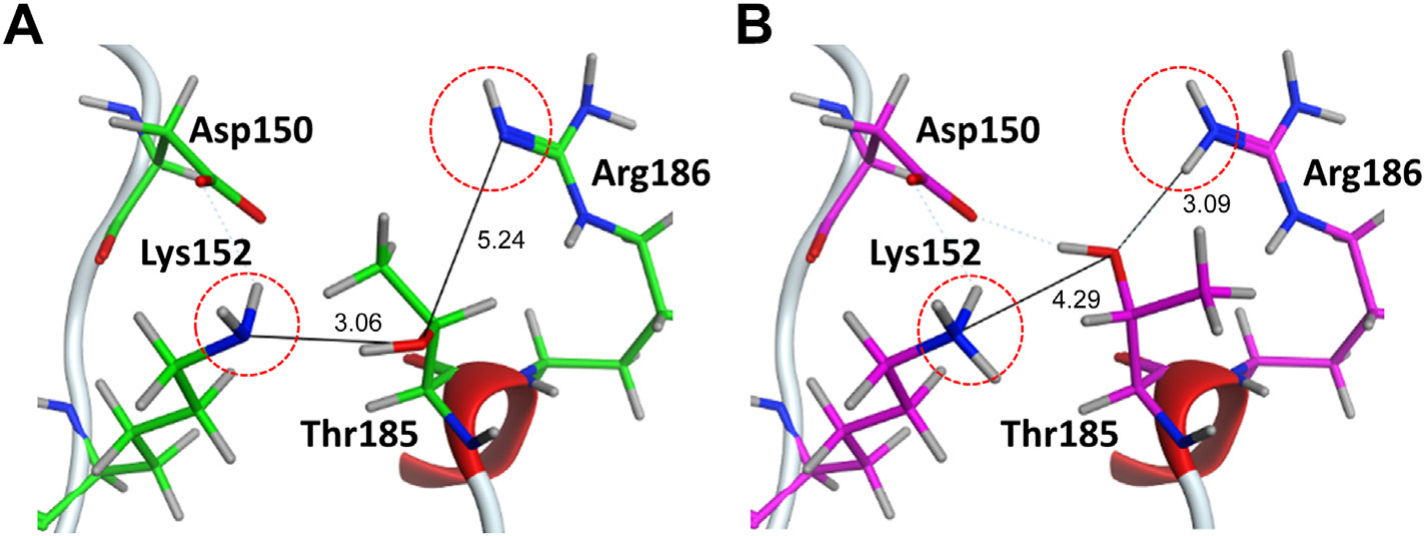
Comparison of the 3D structure of the complemented and non-complemented regions with hydrogen bonds at the Asp150, Lys152, Thr185, and Arg186 residues (PDB ID: 3ROC): (A) complemented structure and (B) non-complemented structure. Numerals denote the interatomic distances in units of Å.

Next, we discuss the origin of the difference of about 10 kcal/mol [= (16.2–5.31) kcal/mol] in the binding energy other than the contribution of missing residues. The example structure 3ROC has 11 missing amino acid residues consisting of sequences from Ala172 to Tyr182. Table 3 shows the IFIE values affected by the difference of protonation state between complement and non-complemented structures in this region. First, from Fig. 11, we note that the protonation states of Lys118 in complemented and non-complemented structures are deprotonated and protonated states, which are the neutral and charged states, respectively. The difference of protonation states affected the IFIE of ligand with Lys118 and those around them by about several kcal/mol. To understand the other causes of the difference in IFIE between completed and non-completed structures, we show the protein structure around the Thr185 residue in Fig. 12. The side chain of Thr185 in 3ROC has missing atoms. The orientation of Thr185 is different in the complemented and non-complemented loop structures. The protonation states of the Lys152 and Arg186 residues, which are close to Thr185, changed so that these residues formed hydrogen bonds with different orientations with respect to Thr185 (Table 3). However, as shown in Fig. 11, Fig. 12, the remaining missing groups influenced the other remaining groups and unrelated segments of the protein.

**Table 3:** Comparison of IFIEs (in kcal/mol) for amino acid residues with different protonation states (PDB ID: 3ROC).

Amino acid residue	IFIEs of ligand [kcal/mol]		
	Complement (Structure A)	Non-complement (Structure B)	Diff
Lys118	0.38	3.28	−2.90
Tyr182	0.34	n/a^a^	0.34
Val183	−0.30	−0.01	−0.29
Asp150	−3.02	−2.61	−0.41
Lys152	0.35	3.81	−3.46
Thr185	0.15	0.16	−0.01
Arg186	0.08	1.76	−1.68

a n/a denotes data not available because the residue is missing.

#### 3.2.4 Influence of different force fields on the IFIE values

**Fig. 13:**
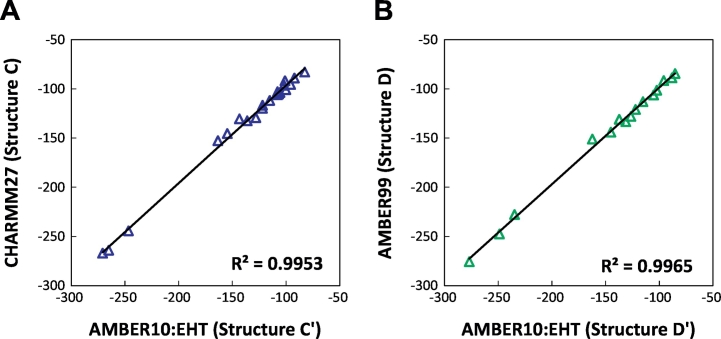
Correlations of IFIE-sums obtained with different force fields: (A) CHARMM27 versus AMBER10:EHT (Structure C versus C′) and (B) AMBER99 versus AMBER10:EHT (Structure D versus D′).

**Fig. 14:**
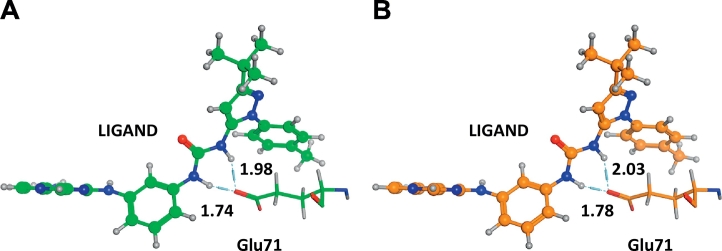
The distances (in units of Å) between the hydrogen atoms in urea group of ligand and the oxygen atom in carboxyl group of Glu71 for the structures C′ and C (PDB ID: 3GCU). Structure C′and C are shown in (A) and (B), respectively.

**Fig. 15:**
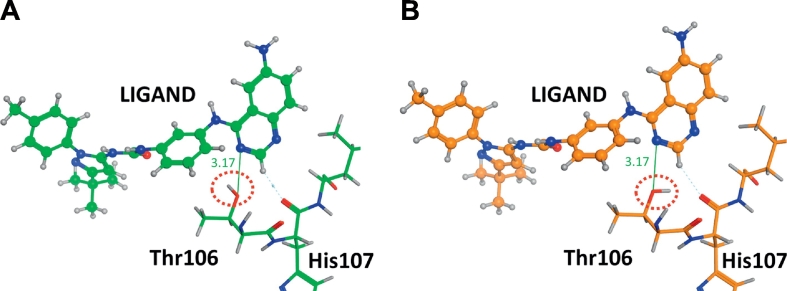
The orientation of hydrogen atoms of side chain on Thr106 near ligand for the structures C′ and C (PDB ID: 3GCU). (A) Structure C′ shows that the hydrogen atom of the hydroxyl group of Thr106 is directed towards the benzene ring of ligand. (B) Structure C shows that the hydrogen atom of the hydroxyl group of Thr106 is directed towards the His107 residue.

We employed three force fields, AMBER10:EHT, AMBER99, and CHARMM27, to investigate the influence of the force field used for the geometry optimization upon IFIE-sum on ligand. The IFIE-sum obtained with AMBER10:EHT was used as a reference for comparison. Fig. 13 shows that using a different force field for geometry optimization has little effect on the ultimate IFIE-sum. The IFIE-sums obtained with each force field are listed in Table 4, and no significant differences are observed with certain exceptions. To understand the origin of the exceptions, we also list the residues with the largest differences in IFIE between force fields in Table 5. This table says Glu71 and Thr106 frequently provide large contributions to the difference in IFIE-sum. As shown in Fig. 14, the Glu71 and the urea type ligands make the hydrogen bonds. The differences in IFIE of Glu71 become larger especially in the cases of the urea type. The structures around Glu71 and the urea-type ligand were, however, almost unchanged by difference of force field (Fig. 14). This would be explained by the fact that the IFIE values between the urea-type ligands and Glu71 were large negative values (from −45 to −60 kcal/mol) in comparison to those of other fragment pairs. These values were sensitive to the slight structural difference of hydrogen atoms due to the different force fields. This sensitivity may be related to the poor correlation between IFIE and pIC_50_ for the urea type (Fig. 7D). Next, the 3D structures around Thr106 minimized by using different force fields are shown in Fig. 15, where 3GCU is employed as an example. In structure C′ with AMBER10:EHT force field, the OH group in the side chain of Thr106 faces the ligand, while in structure C with CHARMM27 force field, it faces the main chain of His107. Optimizing the structures under the different conditions to determine the direction of the OH group, we found that the final direction was determined by its initial position.

**Table 4:** Comparison of IFIE-sums (kcal/mol) obtained with different force fields. Comparison between CHARMM27 and AMBER10:EHT (top) and comparison between AMBER99 and AMBER10:EHT (bottom). Difference of IFIEs are shown by absolute values.

PDB ID	IFIE-sum [kcal/mol]			Ligand charge	DFG-loop	Ligand structure types
	CHARMM27 (Structure C)	AMBER10:EHT (Structure C′)	Diff.			
3GCU	−130.52	−143.30	12.78		out	Urea
3GCQ	−152.72	−163.36	10.64		out	Urea
1BMK	−91.69	−100.86	9.16		in	Three aromatic ring linked
3GCV	−145.35	−154.51	9.15		out	Urea
4KIQ	−96.37	−101.72	5.35		in	Others
3HV7	−116.63	−121.77	5.14		out	Urea
4KIN	−102.90	−107.84	4.94		in	Others
3HEC	−267.07	−270.74	3.67	+	out	Others
3DS6	−132.57	−136.03	3.46		in	Biphenyl amide
3O8T	−111.74	−115.12	3.38		out	Urea
3C5U	−88.95	−92.11	3.16		in	Three aromatic ring linked
4A9Y	−244.38	−246.69	2.32	+	out	Others
3D7Z	−105.99	−108.16	2.17		in	Biphenyl amide
4KIP	−119.80	−121.83	2.03		in	Others
3OC1	−103.69	−105.64	1.95		out	Urea
3O8U	−105.91	−107.77	1.86		out	Urea
3IPH	−105.15	−106.76	1.61		in	Urea
3HV4	−129.24	−128.06	1.18		out	Biphenyl amide
1BL7	−263.95	−265.04	1.08	+	in	Three aromatic ring linked
2ZB0	−100.79	−99.99	0.81		in	Biphenyl amide
3HP5	−82.94	−82.46	0.47		in	Others
3ZSI	−95.52	−95.60	0.08		out	Others


PDB ID	IFIE-sum [kcal/mol]			Ligand charge	DFG-loop	Ligand structure types
	AMBER99 (Structure D)	AMBER10:EHT (Structure D′)	Diff.			
3PG3	−151.01	−162.31	11.29		out	Urea
3MW1	−227.70	−234.81	7.11	+	in	Others
3HV3	−130.91	−137.29	6.38		out	Urea
3OCG	−91.53	−95.85	4.32		in	Others
3O8P	−133.38	−131.16	2.23		out	Urea
3OBJ	−113.10	−115.23	2.13		out	Urea
3ZSG	−128.01	−126.54	1.47		out	Three aromatic ring linked
3HV6	−275.65	−276.99	1.34	+	out	Urea
2BAK	−247.53	−248.86	1.33	+	out	Others
3HEG	−143.96	−145.15	1.19		out	Urea
3D83	−121.04	−122.17	1.13		out	Biphenyl amide
2ZB1	−84.44	−85.36	0.92		in	Biphenyl amide
2BAJ	−101.46	−102.35	0.90		out	Urea
1ZZL	−106.23	−105.36	0.87		in	Three aromatic ring linked
3FC1	−88.78	−88.18	0.60		in	Others

**Table 5:** List of the amino acid residues with largest differences in IFIE between different force fields: CHARMM27 and AMBER10:EHT (top), and AMBER99 and AMBER10:EHT (bottom), where the IFIE values were evaluated using structure C′ or structure D′.

IFIEs of AMBER10:EHT and CHARMM27					
PDB ID	Ligand structure types		Order of Diff.^b^		
			1st	2nd	3rd
3GCU	Urea	Residue	**Thr106**	**Glu71**	Thr68
		IFIE (reference)^a^	**−8.71**	**−50.42**	−3.04
		Diff.^b^	**7.77**	**2.70**	0.90
3GCQ	Urea	Residue	**Thr106**	**Glu71**	Leu108
		IFIE (reference)^a^	**−6.24**	**−63.39**	−8.37
		Diff.^b^	**5.53**	**4.70**	1.06
1BMK	Three aromatic ring linked	Residue	Gly110	Lys53	Met109
		IFIE (reference)^a^	−9.97	−38.58	−7.98
		Diff.^b^	4.72	3.26	3.13
3GCV	Urea	Residue	**Glu71**	Met109	Ala172
		IFIE (reference)^a^	**−48.49**	−11.66	−2.95
		Diff.^b^	**4.41**	3.03	2.55
4KIQ	Others	Residue	**Glu71**	Asp168	Phe169
		IFIE (reference)^a^	**−24.22**	6.64	−6.34
		Diff.^b^	**4.81**	1.06	1.04
3HV7	Urea	Residue	**Glu71**	Asp168	Lys53
		IFIE (reference)^a^	**−47.85**	−14.96	1.03
		Diff.^b^	**2.62**	1.82	1.55

IFIEs of AMBER10:EHT and AMBER99					
PDB ID	Ligand structure types		Order of Diff.^d^		
			1st	2nd	3rd
3PG3	Urea	Residue	Leu74	**Glu71**	Arg70
		IFIE (reference)^c^	−1.35	**−60.66**	−6.23
		Diff.^d^	6.42	**3.92**	0.89
3 MW1	Others	Residue	Met109	Gly110	Asp168
		IFIE (reference)^c^	−5.89	−11.23	−81.83
		Diff.^d^	4.12	2.83	0.61
3HV3	Urea	Residue	Met109	**Glu71**	Gly31
		IFIE (reference)^c^	−4.26	**−55.56**	−6.87
		Diff.^d^	4.72	**1.66**	1.58

a IFIE values of Structure C′ (AMBER10:EHT).

b Absolute value of difference on IFIEs between Structures C′ (AMBER10:EHT) and C (CHARMM27).

c IFIE values of Structure D′ (AMBER10:EHT).

d Absolute value of difference on IFIEs between Structures D′(AMBER10:EHT) and D (AMBER99).

We conclude that the difference in IFIE-sum between different force fields for geometry optimization was almost negligible by 5 kcal/mol or less as far as starting from X-ray crystallographic atomic coordinates, specifically when the structures were optimized into the same local stable geometry; the dependency on force field appears in IFIE modulation for length of hydrogen bonds and orientation of hydroxyl groups.

## 4 Conclusion

In this study, we performed an FMO-IFIE analysis of the intermolecular interactions between the amino acid residues of the p38 MAP kinase and its inhibitors. First, a good correlation between the experimental IC_50_ values and the calculated IFIE-sums for neutral ligands and proteins in the DFG-in conformation was obtained, in contrast to that of charged ligands and DFG-out conformations, where the IFIE values between the ligands and Glu71 caused the poor correlation between the IFIE-sum and pIC_50_. Moreover, when different types of ligands were bound to the protein, the correlation coefficients were moderate except for urea ligands, thus suggesting that the type of ligand also affects the calculated IFIEs.

In addition, the correlation coefficients varied by 0.23 when different approaches to complementing the missing residues were used. This difference is composed of the contribution from complemented residues as well as change of protonation state or change in hydrogen orientation. Furthermore, the difference in IFIE obtained with different force fields was less than 5 kcal/mol, except for some cases, when the same local minimum energy structures were used. One of the reasons for the exceptions was that the IFIEs between Glu71 and the urea type ligands were significantly higher than those of other fragment pairs. This results in fluctuating tendency of IFIEs between Glu71 and the urea-type ligands due to the slight difference in position of hydrogen atom. The other reasons were that complementing the missing region or adding hydrogen atoms might change the hydrogen orientation or protonation state and thus cause a difference in IFIE. Although this difference did not make significant effects for main conclusion of this research, this potential for different protonated state caused by difference of modeling procedure may produce large differences in other research objects. Thus, sufficient care should be taken for modeling around ligand pocket.

This study provides a practical way to understand the relationship between different preprocessing procedures and the calculated IFIE values of the protein-ligand complexes and thus to achieve a good correlation between calculation and experiment. We expect that these results will contribute to the practical application of the FMO method to drug design in pharmaceutical industries. In addition, this research was limited to discuss the choice of preparation procedure, or the correlation between experimental IC_50_ and FMO derived IFIE-sum for some parts of PDB structures. We did not use more theoretically sophisticated approaches such as informatics approaches, for example, singular value decomposition [24] or physicochemical approaches especially for incorporation of solvation effects: FMO based polarizable continuum model (FMO-PCM) [25], FMO based Poisson–Boltzmann surface area (FMO-PBSA) [26,27] and FMO method with molecular mechanics Poisson–Boltzmann surface area (FMO+MM-PBSA) [10], because these methods require more computation resources; for example, FMO-PBSA requires the computational time by about 20 times. Furthermore, we discussed neither the efficacy of FMO3 method [8,28], which would be important for bridging water between protein and ligand [29], nor the effects of structural sampling. These more sophisticated approaches, including higher-order electron correlation methods and higher basis sets (for example, cc-pVDZ) [[30], [31], [32]], may be promising to improve the correlation between the calculated IFIE values and experimental pIC_50_ data.

## Appendix A Details of structure preparation

Details of the preparation procedure for each structure are shown below.

Structure A (built by institution “a” mainly with MOE)

(1) Obtaining X-ray structures
  Original X-ray structures were downloaded from the PDB repository.
(2) Sequences for calculations
  We used amino acid residues #6-351 for the calculations because in this region, the atomic coordinates of all the structures were determined by X-ray crystallography.
(3) Missing residues
  Missing residues were complemented with the “Complement Main Chain” function in BioStation Viewer. We employed PDB structures 3GC7 and 3D83 as template structures for the DFG-in conformation and DFG-out conformation, respectively, because these structures have no missing residue and exhibit the best resolution of 1.7 Å (3GC7) and 1.9 Å (3D83).
(4) Missing atoms
  Missing atoms were complemented with the “Structure Preparation” function in MOE.
(5) Setting the bond order and ionization of the ligand
  We fixed bond orders and ionization of ligand according to the 2D chemical structures.
(6) Crystal water molecules
  All crystallographic water molecules were removed except for the water molecule forming a hydrogen bond with Asp168 and Lys53 in the DFG-in conformation and the water molecules forming a hydrogen bond with Asp168 in the DFG-out conformation.
(7) Hydrogen atom addition
  We used the “Protonate3D” function in MOE for pH = 7.0. In addition, the N-terminus and C-terminus are NH_3_^+^ and COO^−^, respectively.
(8) Tautomeric states and protonation of histidine
  We dealt with His312 as HIP. Other His residues were treated as neutral residues. The tautomeric states of His such as HIE or HID were determined with Protonate3D in MOE.
(9) Assignment of ligand charge
  We assigned ligand charges according to their protonation state at pH = 7.0 in water. For aliphatic amines, we set the formal charges to +1. For carboxylic groups, we set the formal charge to −1, and the formal charge of aromatic groups such as anilines was set to zero.
(10) Structure optimization.
  For the geometry optimization, the heavy atoms whose coordinates were determined by X-ray crystallography were fixed; the hydrogen atoms used to complement the missing residues and missing atoms were not fixed. MOE with the AMBER10:EHT force field was used.

Structure B (built by institution “a” with MOE; structure with non-complementation for missing residues)

(1) Obtaining X-ray structures
  Same as structure A.
(2) Sequences for calculations
  Same as structure A.
(3) Missing residues
  We did not complement missing residues. For the C-terminus, we employed COOH and for the N-terminus, we employed NH_2_.
(4) Missing atoms
  Same as structure A.
(5) Setting the bond order and ionization of the ligand
  Same as structure A.
(6) Crystal water molecules
  Same as structure A.
(7) Hydrogen atom addition
  Same as structure A.
(8) Histidine tautomeric states
  Same as structure A.
(9) Assignment of ligand charge
  Same as structure A.

Structure C built by institution “b” and “c” mainly with Discovery Studio using CHARMM27 force field)

(1) Obtaining X-ray structures
  Same as structure A.
(2) Sequences for calculations
  Same as structure A.
(3) Missing residues
  First, missing residues were complemented with the “Complement Main Chain” function in BioStation Viewer. If the structures were significantly distorted or deeply contacted, we complemented the missing residues with Discovery Studio.
(4) Missing atoms
  We complemented the missing atoms with Discovery Studio
(5) Setting the bond order and ionization of the ligand.
  Same as structure A.
(6) Crystal water molecules
  Same as structure A.
(7) Hydrogen atom addition
  Similar to structure A. Here, “GBpK” function in Discovery Studio was employed instead of “Protonate3D” function in MOE.
(8) Histidine tautomeric state
  Similar to structure A. Here, “GBpK” function in Discovery Studio was employed instead of “Protonate3D” function in MOE.
(9) Assignment of ligand charge
  Same as structure A.
(10) Structure optimization
  The optimization conditions were the same as for structure A. The CHARMM27 force field of Discovery Studio was employed for the optimization.

Structure C′(similar to structure C and hydrogen atoms were reoptimized with AMBER10:EHT force field)

(1) to (10)
  Same as structure C.
(11) Structure optimization (II)
  We used AMBER10:EHT for the optimization of structure C for hydrogen atoms.

Structure D (built by institution “d” mainly with MOE using Amber99 force field; MOE was partly employed for the complementation of missing residues.)

(1) Obtaining X-ray structures
  Same as structure A.
(2) Sequences for calculations
  Same as structure A.
(3) Missing residues
  1) If the overlaps with the template were large, we copied a template structure using MOE.
  2) If the difference in the conformations of the activation loop were too large, we used the “Build loop” function of MOE.
(4) Missing atoms

Same as structure A (Structure Preparation in MOE).

(5) Setting the bond order and ionization of the ligand

Same as structure A.

(6) Crystal water molecules

Same as structure A.

(7) Hydrogen atom addition

Same as structure A.

(8) Histidine tautomeric states

Same as structure A.

(9) Assignment of ligand charge

Same as structure A.

(10) Structure optimization
  The optimization conditions were the same as for structure A. The AMBER99 force field of MOE was employed for the optimization.

Structure D′ (similar to structure D and hydrogen atoms were reoptimized with AMBER10:EHT force field)

(1) to (10)
  Same as structure D.
(11) Structure optimization (II)

We used AMBER10:EHT for the optimization of structure D for hydrogen atoms.
